# A Curious Case of Intraductal Papillary Mucinous Neoplasm of the Bile Duct Presenting With Cholangitis

**DOI:** 10.7759/cureus.101759

**Published:** 2026-01-17

**Authors:** Raghavendra Rao Rachapoodi Venkata, Panduranga Rao Kondadasula, Krishna Kalyan Reddy Janumpalli

**Affiliations:** 1 Department of Surgical Gastroenterology and Liver Transplantation, National Institute of Gastroenterology and Liver Diseases (Renova-NIGL), Hyderabad, IND; 2 Department of Medical Gastroenterology and Hepatology, National Institute of Gastroenterology and Liver Diseases (Renova-NIGL), Hyderabad, IND

**Keywords:** biliary cystic neoplasm, cholangitis, ipmn-b, ipnb, premalignant biliary lesions

## Abstract

Intraductal papillary mucinous neoplasm of the bile duct (IPMN-B) belongs to a group of biliary disorders that are premalignant lesions and have a relatively low incidence. We describe a case of a patient with IPMN-B who presented unusually with cholangitis and underwent surgical resection as a definitive treatment. The patient was a 65-year-old male who presented with a six-month history of recurrent right upper abdominal pain and loss of appetite, with a recent history of jaundice. Blood investigations showed leukocytosis with altered liver function test, with elevated bilirubin and liver enzymes. Ultrasound of the abdomen showed features suggestive of a cystic lesion in the left lobe of the liver. Further evaluation with a contrast MRI with magnetic resonance cholangiopancreatography (MRCP) showed a cystic lesion measuring 4x4 cm with mural lesions and biliary communication to the left hepatic duct. Serum CA19-9 was within normal limits (2 U/mL). Patient underwent endoscopic retrograde cholangiopancreatography (ERCP) in view of cholangitis and later had surgery in the form of left lateral sectionectomy with intraoperative cholangioscopy. The postoperative period was uneventful, and the patient was discharged on the fourth postoperative day. The histopathology report showed features suggestive of intraductal papillary mucinous neoplasm of the biliary tract with low-grade dysplasia. Patient was followed up at three months and was found to be without any complications. IPMN-B is a rare disease, and patients presenting with cholangitis are rather uncommon. Physicians need to be aware of such cases, as their management is different from cholangitis due to other causes, which are frequently encountered in clinical practice. IPMN-B requires a detailed imaging workup and definitive surgical resection due to its high risk of malignant transformation.

## Introduction

Intraductal papillary mucinous neoplasms of the bile duct (IPMN-B) represent a group of premalignant biliary lesions with a relatively low incidence [1]. They usually present as complex cystic lesions that require multidisciplinary evaluation for effective management. IPMN-B typically presents insidiously with nonspecific clinical and radiological findings. We present a case of a 65-year-old male who developed cholangitis and was subsequently diagnosed with IPMN-B following surgical resection, accompanied by a brief review of intraductal papillary neoplasm of the bile ducts (IPNBs) and their management.

## Case presentation

A 65-year-old male presented with a six-month history of recurrent right upper abdominal pain and loss of appetite. He reported a recent exacerbation of pain associated with fever and jaundice. The patient had a significant history of tobacco chewing. Clinical examination revealed good performance status, icterus, and mild tenderness in the right hypochondrium. Laboratory investigations demonstrated leukocytosis and altered liver function tests (Table 1).

**Table 1:** Laboratory investigations of the case. ALT: alanine aminotransferase; AST: aspartate aminotransferase; ALP: alkaline phosphatase; GGTP: gamma-glutamyl transpeptidase

Test	Patient value	Reference range
Total leukocyte count	11,630 cells/mm³	4,000-10,000 cells/mm³
Total bilirubin	2.2 mg/dL	0.2-1.3 mg/dL
Direct bilirubin	1.4 mg/dL	0.1-0.25 mg/dL
ALT	144 U/L	13-69 U/L
AST	129 U/L	15-46 U/L
ALP	431 U/L	38-126 U/L
GGTP	518 U/L	12-58 U/L

**Figure 1:**
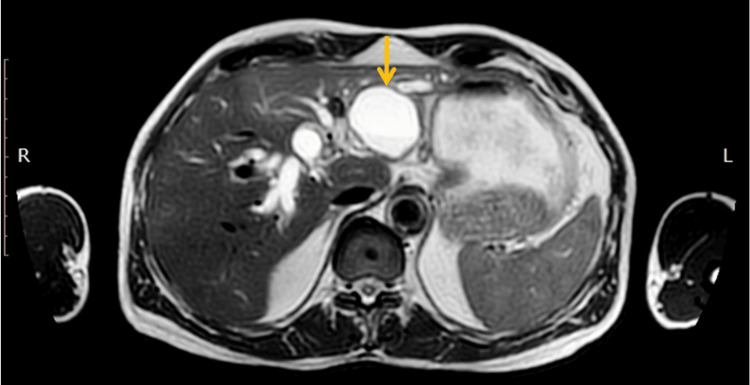
T2 axial section of MRI abdomen. Cystic lesion (arrow) in segment II-III with bilateral intrahepatic biliary duct dilatation.

**Figure 2:**
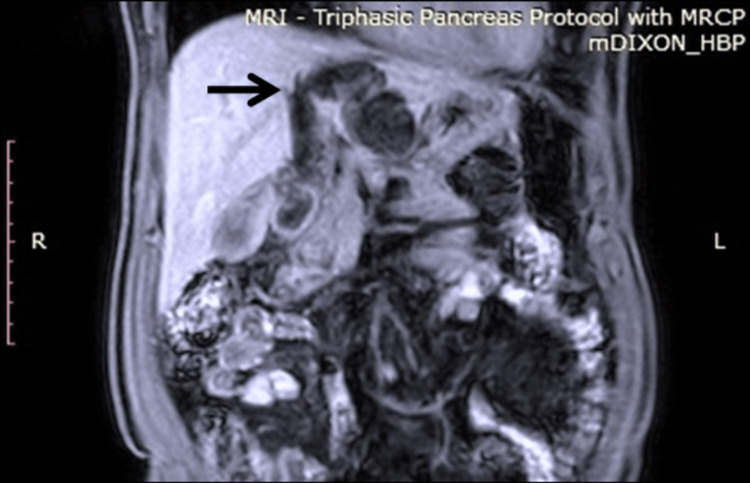
T1 Dixon coronal section of MRI abdomen. The cystic lesion is noted to have communication (arrow) to the dilated left hepatic duct.

**Figure 3:**
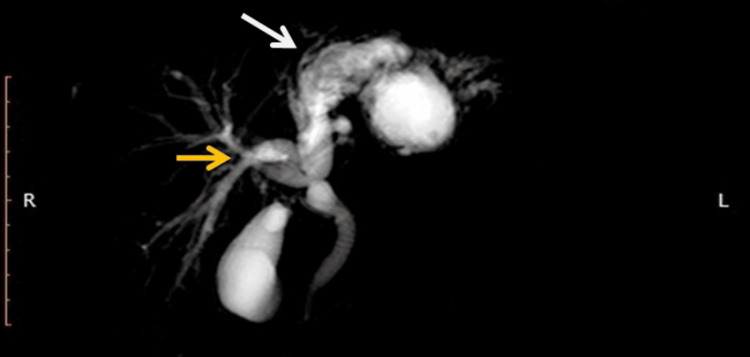
MRCP reconstruction image. The cystic lesion is in continuity (white arrow) with the left hepatic ductal system, along with right hepatic ductal system dilatation (orange arrow). MRCP: magnetic resonance cholangiopancreatography

Abdominal ultrasonography revealed a cystic lesion in the left lobe of the liver with dilation of the intrahepatic bile ducts (IHBD) and common bile duct (CBD). Further evaluation with contrast-enhanced magnetic resonance imaging (MRI) and magnetic resonance cholangiopancreatography (MRCP) identified a 4×4 cm cystic lesion with mural nodules and communication with the left hepatic duct (Figures 1-3).

**Figure 4:**
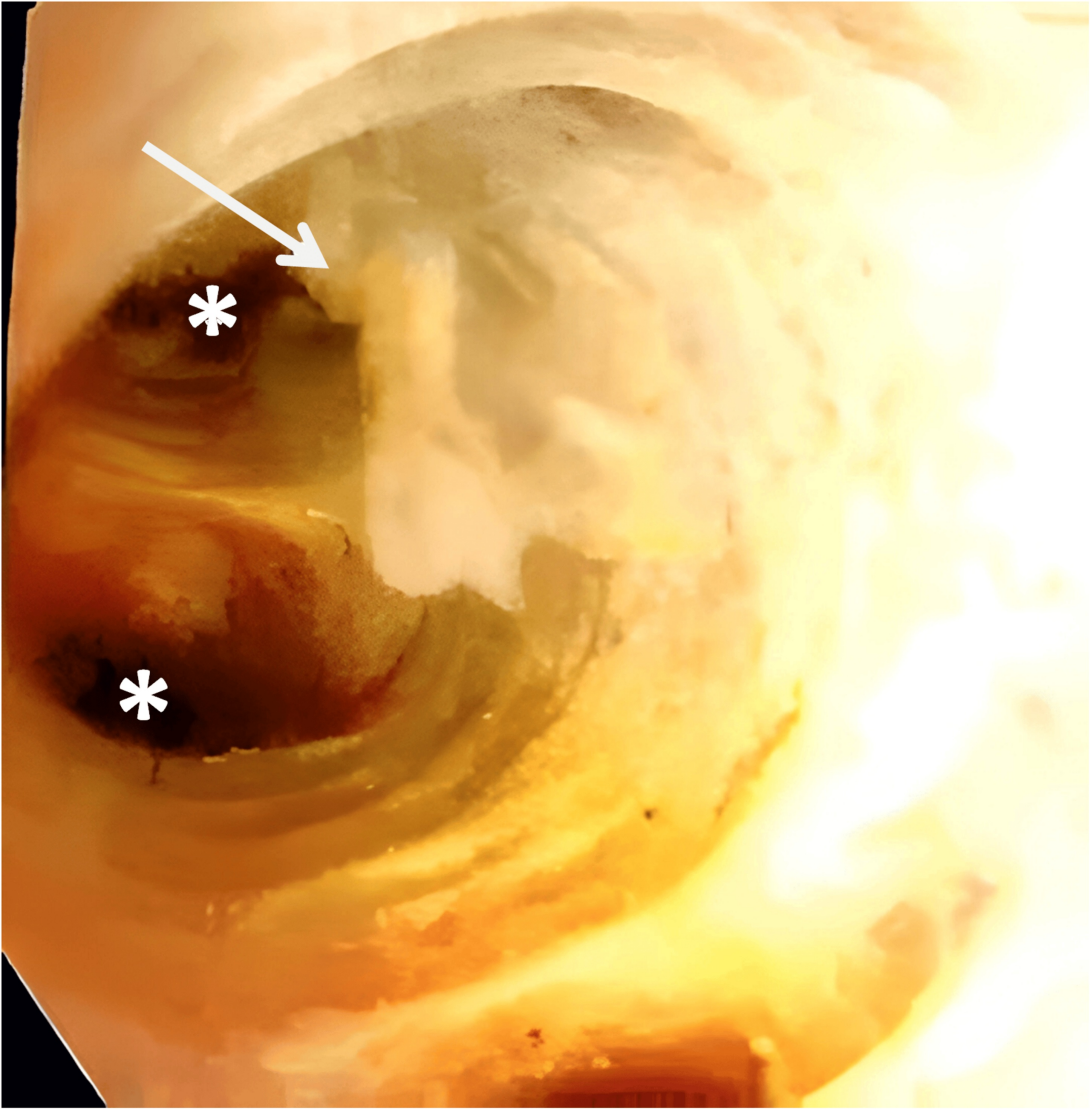
Intraoperative cholangioscopy Mucinous material (arrow) is noted within the bile ducts along with visualization of openings (asterisk) of the intrahepatic bile ducts.

**Figure 5:**
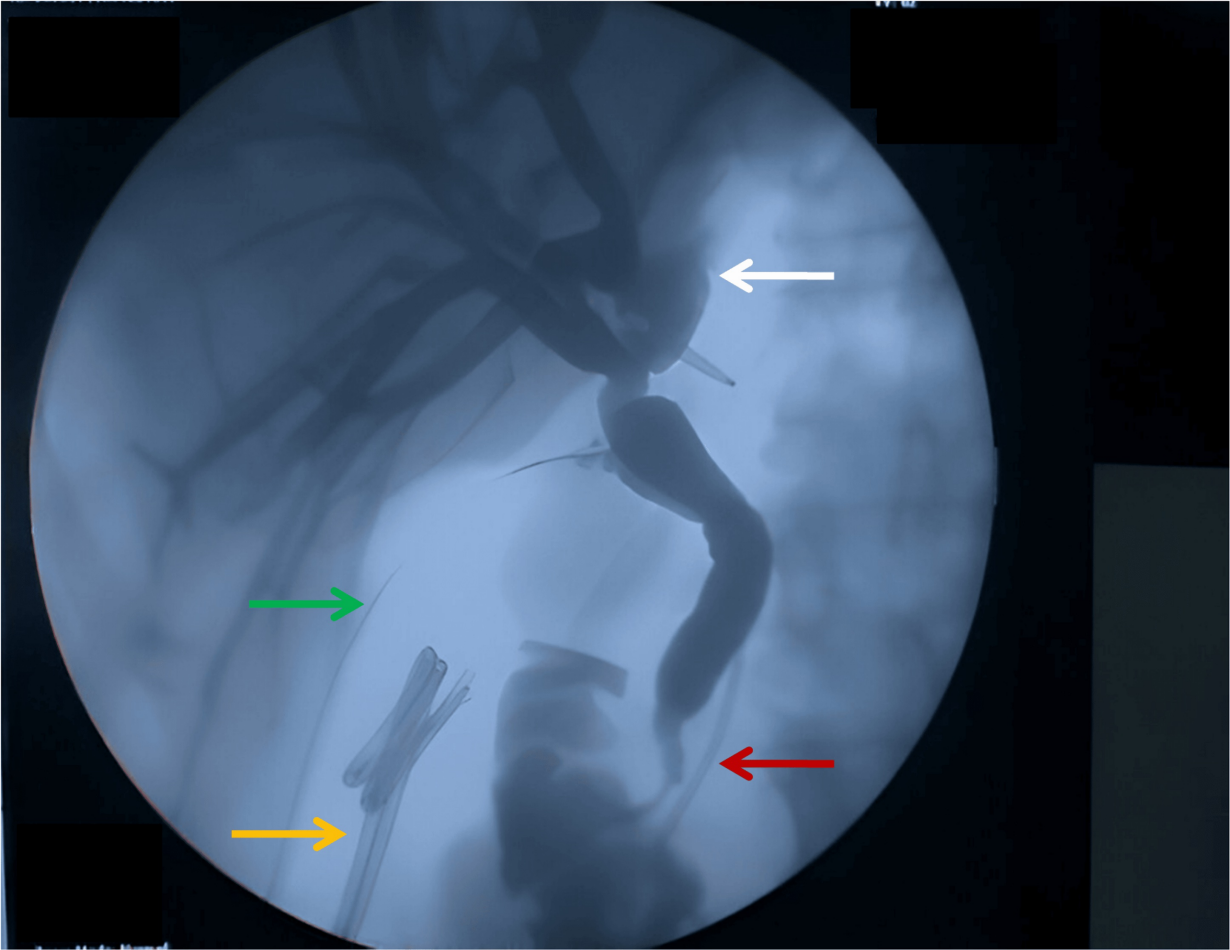
Postresection cholangiogram. Cholangiography shows complete resection of the lesion with no obvious contrast leak at the remnant left hepatic duct stump (white arrow). Passed off biliary stents (orange arrow) are noted in the colonic lumen. A pancreatic ductal stent is noted in situ (red arrow). A cholangiogram catheter is noted passing through the cystic duct stump (green arrow).

Serum CA19-9 levels were within normal limits (2 U/mL). The patient underwent endoscopic retrograde cholangiopancreatography (ERCP) in view of cholangitis, which revealed mucin-like material within the biliary tract, and CBD stenting was performed. Subsequently, a left lateral sectionectomy with intraoperative cholangioscopy was performed via a laparotomy. The cystic lesion demonstrated biliary communication with the segment II-III duct, and mucin-like material was observed throughout the bile ducts. Intraoperative cholangioscopy confirmed the presence of mucin and revealed no additional mural lesions within the intrahepatic ducts (Figure 4). Contrast cholangiography was performed intraoperatively to assess the completeness of resection and detect potential biliary leaks (Figure 5).

**Figure 6:**
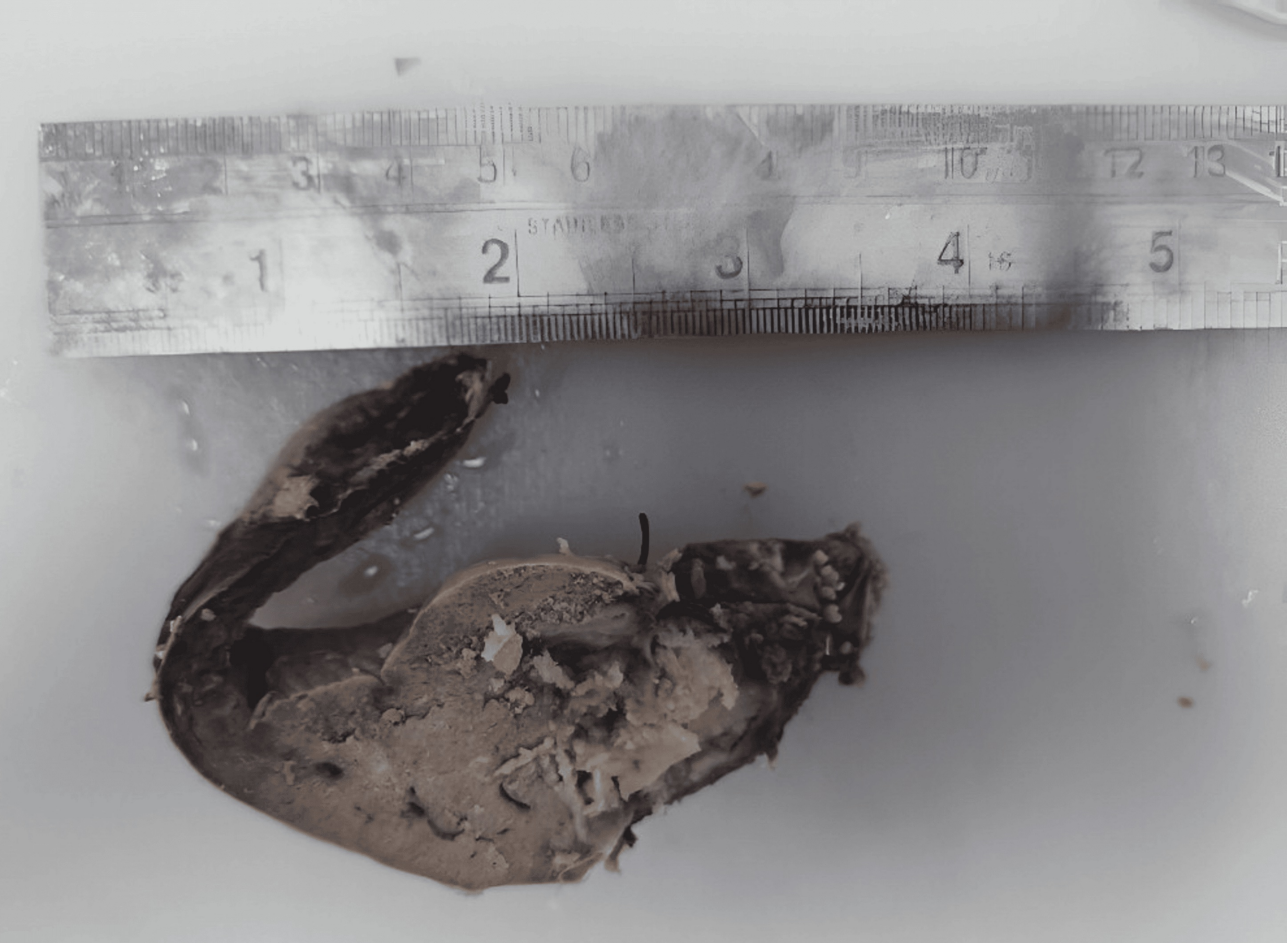
Gross specimen. A papillary tumor was noted within the dilated bile duct.

**Figure 7:**
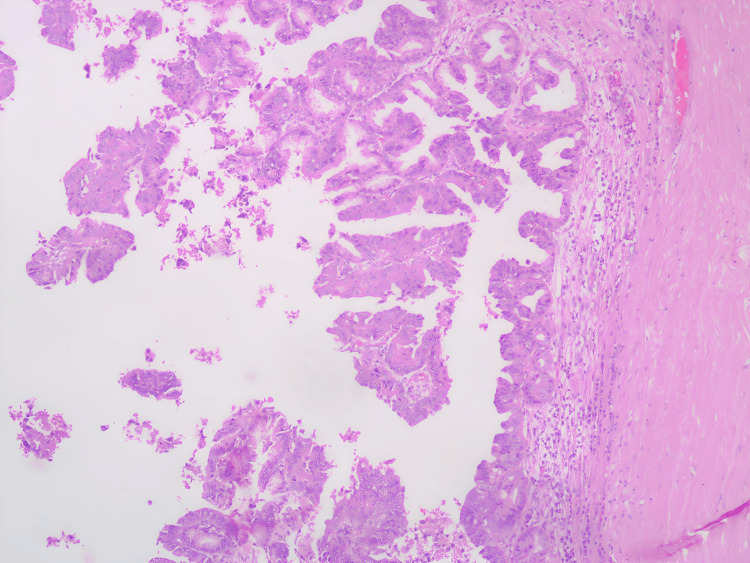
Histopathological examination (HPE) scanner view. Section showing papillary architecture with cyst wall.

**Figure 8:**
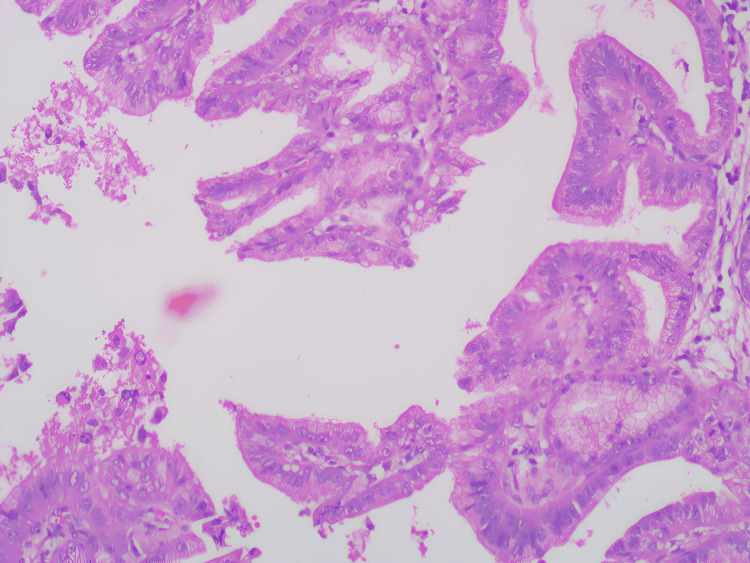
Histopathological examination (HPE) low power view. Section showing papillae with a mucinous type of epithelium of pancreatobiliary type histology.

**Figure 9:**
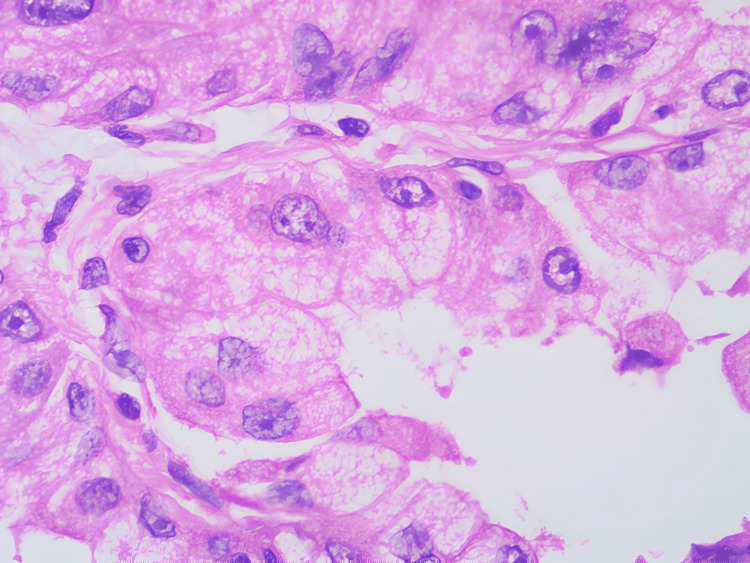
Histopathological examination (HPE) high power view. Section showing cells with pleomorphism and atypia.

The postoperative course was uneventful, and the patient was discharged on the fourth postoperative day. Histopathology analysis revealed features consistent with intraductal papillary mucinous neoplasm of the biliary tract, pancreatobiliary type, with low-grade dysplasia (Figures 6-9). Margins were reported to be negative. At the three-month follow-up, the patient remained in good health.

## Discussion

The World Health Organization (WHO) Classification of Tumours of the Digestive System, published in 2010, proposed that the disease entity previously designated as "biliary cystadenoma/adenocarcinoma" should instead be classified as either intraductal papillary neoplasm of the bile duct (IPNB) or mucinous cystic neoplasm (MCN), depending on the presence of bile duct communication (BDC) and ovarian-like stroma [1]. Cystic neoplasms of the liver are classified as MCN if they exhibit ovarian stroma and do not have BDC, whereas they are classified as IPNB if they demonstrate BDC without ovarian stroma. However, there have been case reports of IPNB with unclear BDC [2], MCN with biliary communication [3], and instances in which both ovarian stroma and BDC are absent [4]. These variations contribute to the complexity of already challenging diagnostic criteria. Further research is warranted to clarify whether a third subtype of cystic neoplasm exists, characterized by mucinous epithelium in which both ovarian stroma and BDC are absent [4].

IPMN-B is a rare biliary tract tumor categorized under mucin-secreting tumors of IPNB, according to the latest WHO classification of biliary tract tumors [5]. IPNBs account for approximately 10% of all bile duct tumors and are more commonly observed in males in the fifth and sixth decades of life. They are more frequently reported in eastern countries where hepatolithiasis and clonorchiasis are endemic [6]. Other risk factors described in the literature include primary sclerosing cholangitis, immunoglobulin G4 (IgG4)-related sclerosing cholangitis, and cholangiectatic anomalous bile ducts [7]. Two variants of IPNBs have been described. Type 1 IPNBs are mucin-producing and are designated as IPMN-B due to their similarity to pancreatic IPMNs. Type 2 IPNBs rarely produce mucin and differ variably from pancreatic IPMNs. Other differences between the subtypes are summarized in Table 2.

**Table 2:** Differences between type 1 IPNB and type 2 IPNB. IPNB: intraductal papillary neoplasm of the bile duct; IPMN-B: intraductal papillary mucinous neoplasm of the bile duct

Characteristics	Type 1 IPNB/IPMN-B	Type 2 IPNB
Location	Intrahepatic bile ducts	Extrahepatic bile ducts
Gross features	Cystic dilatation	Fusiform dilatation
Excessive mucin	Frequently seen	Rarely seen
Grade	Mostly high grade with infrequent low/intermediate grade	Always high grade
Histological types	Gastric and intestinal types are more common	Pancreatobiliary and intestinal types are more common
Aggressiveness	Less aggressive	More aggressive
Association with invasive carcinoma	Approximately 50%	>90%
Outcomes	More favorable	Less favorable

Histologically, both types were further divided into the following four subtypes: intestinal, gastric, pancreatobiliary, and oncocytic, with more than one type sometimes co-existing [5,6]. Intrahepatic IPNBs can also be classified morphologically into the following two subtypes: duct-ectatic (main lesion with diffuse cylindrical or fusiform dilatation of bile ducts) and cystic (cystic masses associated with adjacent bile ducts) [8].

Type 2 IPNBs with invasive carcinoma have a higher rate of lymph node metastasis, reflecting their aggressive nature. Patients with intrahepatic IPNBs exhibit more favorable pathological characteristics and postoperative survival outcomes than those with extrahepatic IPNBs [9]. Moreover, the prognosis of invasive carcinoma derived from IPNB is better than that of conventional intrahepatic and extrahepatic cholangiocarcinomas [5,10].

The clinical presentation of IPNBs commonly includes right upper quadrant pain, jaundice, or recurring cholangitis due to biliary obstruction caused by tumor invasion or mucin production; however, some patients may be asymptomatic. The large quantity of mucin secreted in IPMN-B can obstruct the bile ducts and impede biliary drainage, leading to recurrent episodes of cholangitis [11]. Elevated carbohydrate antigen 19-9 (CA 19-9) levels are strongly associated with IPNBs harboring invasive carcinoma. IPNB appears as a single or multiple papillary/polypoid lesions within dilated intra- or extrahepatic bile ducts or peribiliary glands, and may manifest synchronously or metachronously in the biliary tree [12].

Imaging features that raise suspicion for IPNBs include focal extrahepatic or intrahepatic biliary ductal dilatation (with or without biliary wall thickening), intraductal masses or nodules, or growth along the interior ductal wall [13]. Ultrasonography may demonstrate diffuse duct ectasia or localized ductal dilatation. Contrast-enhanced computed tomography (CT) can reveal lesions that are either isodense or hyperdense compared with adjacent parenchyma during the late arterial phase, losing hyperattenuation in the portal venous and delayed phases [14]. MRI provides superior visualization of papillary growth within the bile ducts when compared with CT. T1-weighted MRI typically demonstrates isointense or hypointense lesions, whereas T2-weighted MRI reveals hyperintense masses. MRCP can delineate biliary ductal communication and associated dilatation of extrahepatic and intrahepatic bile ducts. A characteristic feature of IPNBs is the presence of both upstream and downstream bile duct dilatation due to mucin accumulation. Endoscopic examination may reveal a wide patent duodenal papilla or, rarely, extrusion of mucin from the ampulla of Vater [15]. Endoscopic ultrasonography (EUS) enables detailed evaluation of intraductal masses and can be used for guided biopsies and fluid analysis. ERCP is useful for preoperative bile duct decompression, and peroral cholangioscopy (POCS) allows direct visualization of the bile duct and accurate assessment of tumor extent.

Surgical resection remains the mainstay of treatment for IPNB, with the procedure tailored according to tumor location within the biliary tree. Extrahepatic IPNBs may be managed by bile duct resection or pancreatoduodenectomy (Whipple’s procedure), while intrahepatic IPNBs generally require hepatic resection. Intraoperative frozen section analysis of bile duct margins is recommended to confirm complete excision, particularly in cases with ductal spread.

In cases of extensive superficial spread along the bile ducts, even extensive resection may yield positive margins, and liver transplantation with or without pancreaticoduodenectomy may represent the only potentially curative option [16]. In symptomatic patients unfit for surgery, percutaneous cholangiocopic-guided argon plasma coagulation may offer palliative benefit, although evidence supporting this approach is limited [17]. Adjuvant therapy for patients with invasive carcinoma follows standard cholangiocarcinoma guidelines. A meta-analysis revealed that extrahepatic tumor location, type 2 subclassification, R1 resection, elevated CA19-9 level, tumor multiplicity, and adjacent organ invasion are associated with a poorer prognosis [18].

## Conclusions

IPMN-B, classified under IPNB, is a rare disease, and presentation with cholangitis is uncommon. Clinicians should be aware of such cases, as their management differs from that of frequently encountered causes of cholangitis. IPMN-B necessitates a comprehensive imaging evaluation and definitive surgical resection due to its high risk of malignant transformation, which can significantly worsen patient outcomes.
